# Bone Mineral Density and Trabecular Bone Score in Children, Adolescents and Young Adults with Familial Dysautonomia

**DOI:** 10.1007/s00223-025-01400-x

**Published:** 2025-06-24

**Authors:** Bat El Bar-Aluma, Ronit Porges, Liana Tripto-Shkolnik, Shlomit Keler, Noah Gruber, Adi Dagan, Dalit Modan-Moses, Yael Levy-Shraga

**Affiliations:** 1 Faculty of Medical and Health Sciences, Tel Aviv University, Tel Aviv, Israel; 2 Pediatric Pulmonology Unit and Cystic Fibrosis Center, The Edmond and Lily Safra Children’s Hospital, Sheba Medical Center, Tel-Hashomer, Israel; 3 Division of Endocrinology, Diabetes and Metabolism, Sheba Medical Center, Tel Hashomer, Israel; 4 Pediatric Endocrinology and Diabetes Unit, The Edmond and Lily Safra Children’s Hospital, Sheba Medical Center, Tel-Hashomer, Israel

**Keywords:** Familial dysautonomia, Osteoporosis, Bone mineral density, Trabecular bone score

## Abstract

Familial dysautonomia (FD) is characterized by skeletal morbidity, including osteoporosis and increased fracture risk. We aimed to assess bone mineral density (BMD) and trabecular bone score (TBS) in individuals with FD, and to explore correlations with disease severity. This retrospective study included all the patients with FD who performed at least one dual-energy X-ray absorptiometry (DXA) scan at our institution during 2015–2023. Demographic and clinical data obtained from medical records included: medical treatment, anthropometric measurements, Functional Severity Scale (FuSS) score, balance assessment, the Brief Ataxia Rating Scale score, ambulation ability, blood test results and fracture history. Forty-one patients (21 males) had at least one DXA scan**.** The median age at the first scan was 25 years (range 7–47). The mean BMD Z-score was − 1.2 ± 1.5 at the lumbar spine and − 1.3 ± 1.1 at the bilateral proximal femur. The mean TBS Z-score was − 1.8 ± 1.6. The bilateral proximal femur BMD Z-score correlated with better scores of balance (r = 0.612, *p* = 0.001), ambulation (r = 0.627, *p* = 0.001) and ataxia (r = − 0.470, *p* = 0.015). For 67% of the patients, C-terminal telopeptides of type I collagen (CTX) was above the normal range for age. Both CTX and procollagen type I N-terminal propeptide (P1NP) correlated negatively with FuSS (r = − 0.515, *p* = 0.10 and r = − 0.619, *p* = 0.042, respectively) and with L_1–4_ Z-scores (r = − 0.681, *p* = 0.03 and r = − 0.700, *p* = 0.02, respectively). Individuals with FD had low BMD and TBS Z-scores. These parameters were correlated to disease severity, specifically to balance and ambulation. The bone resorption marker was high and negatively correlated with disease severity.

## Introduction

Familial dysautonomia (FD) is an autosomal recessive disorder that disrupts the development and survival of sensory, sympathetic and parasympathetic neurons [1]. The condition is caused by mutations in the *ELP1* gene (formerly known as *IKBKAP*) [2]. FD predominantly affects individuals of Ashkenazi Jewish descent, with an incidence of approximately 1 in 3,703 live births. The disorder presents at birth and progresses throughout life [3]. The clinical manifestations result from severe autonomic and sensory dysfunction that involves most organ systems [1].

The musculoskeletal manifestations of FD include gait disorders, spinal and foot deformities, arthropathies and osteoporosis [4, 5]. Individuals with FD are more likely to have low bone mineral density (BMD). Among individuals with FD whose BMD was determined by dual-energy X-ray absorptiometry (DXA), 90% were reported as having a BMD Z-score < 0; 50% had a Z-score < –2.0 [6]. Factors that may contribute to decreased BMD in the context of FD include impaired mobility, hypotonia, poor nutrition, low body mass index (BMI), and antacid therapy for gastroesophageal reflux. Most patients with FD carry a gastrostomy for nutritional supplementation. However, adequate nutrition is challenged by hyper-adrenergic crises, a cardinal feature of the disease that is characterized by cyclic vomiting and nausea. Moreover, FD predisposes to high resting energy expenditure. Both factors lead to low BMI and consequently decreased BMD [7]. Delayed sexual maturation, which is common in both males and females with FD [8], may also affect BMD. Another factor that may contribute to FD bone disease is the extreme blood pressure fluctuations related to afferent baroreflex failure. These may cause vascular bone disease with hypo-perfusion and bone damage [9].

Data regarding bone quality in FD are sparse. The Elp1 conditional knockout mouse model recapitulated several key hallmarks of human FD [10]. These mice showed significantly reduced whole-bone toughness, and female mice had reduced trabecular microarchitecture but not cortical geometry [11]. The trabecular bone score (TBS) is a textural index that evaluates bone microarchitecture and quality [12]. It is not a direct measurement of bone microarchitecture but reflects three-dimensional bone characteristics, such as trabecular number and separation. TBS is calculated from the spine DXA image and provides complementary information to BMD results. Higher TBS values are associated with more homogeneously structured bone. In adults, TBS has emerged as an important predictor of osteoporotic fractures, independent of major clinical risk factors and BMD [13, 14]. Incorporating TBS into the Fracture Risk Assessment Tool (FRAX) has enhanced fracture risk stratification [15].

**Fig. 1:**
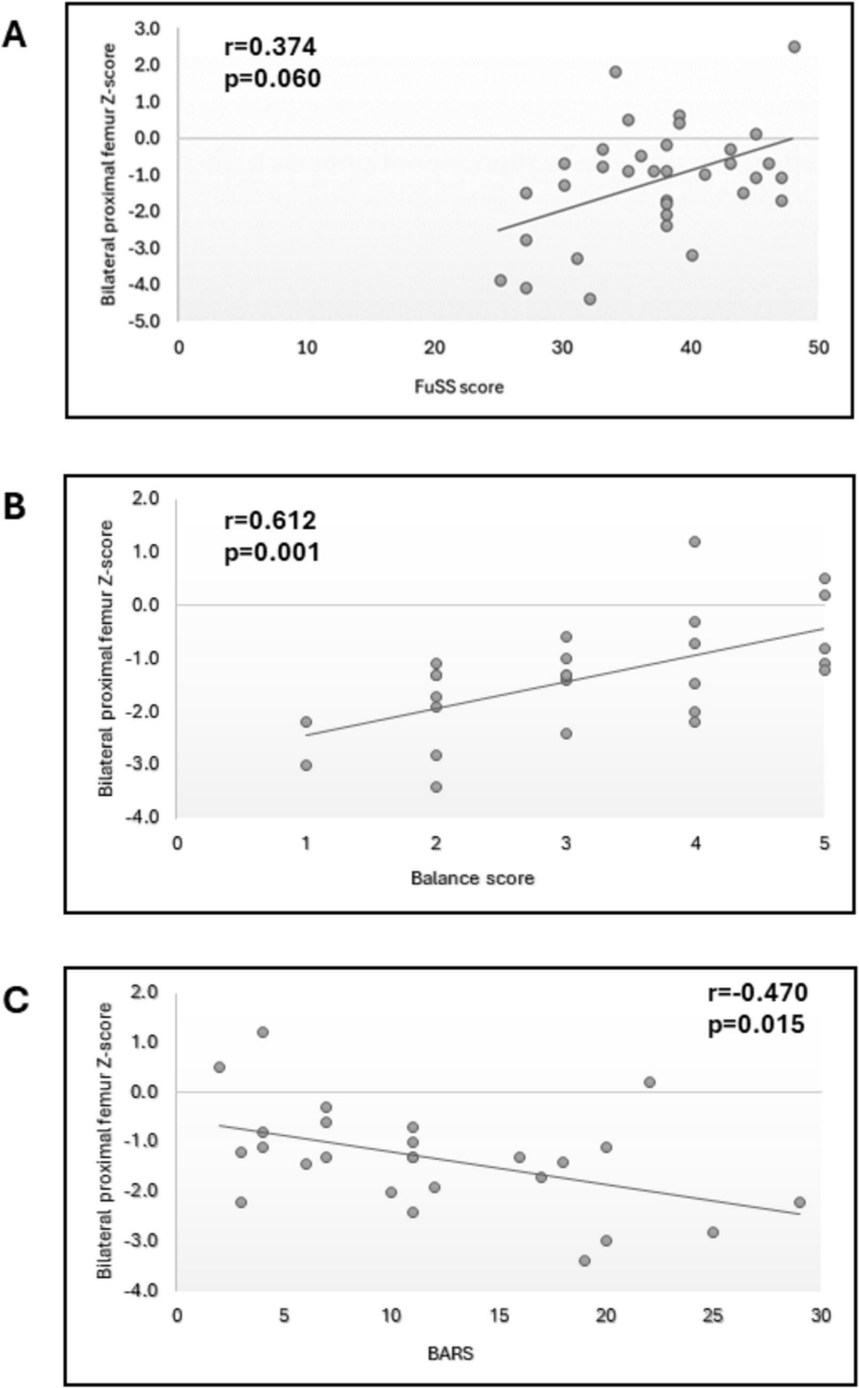
Correlations of bilateral proximal femur Z-scores with the Functional Severity Score (FuSS), balance scores and the Brief Ataxia Rating Scale (BARS)

In recent years, an increasing number of studies have underscored the utility of TBS in the pediatric population [16–19]. Most recently, reference values for TBS in children and adolescents using Hologic densitometers have been published [20]. To the best of our knowledge, bone quality parameters, specifically TBS values, have not been reported for individuals with FD. The objectives of this study were to evaluate BMD and TBS in children, adolescents and young adults with FD, and to explore correlations between BMD and various disease severity scores (Fig. 1).

## Methods

### Study Population

The medical files of all the patients with FD treated at Sheba Medical Center during 2015–2023 were reviewed for this retrospective study. Those who performed at least one DXA scan at our institution during the study period were included in the analysis. We extracted demographic and clinical data from patients' medical records, including: age, gender, anthropometric measurements, fracture history, age at menarche and medical treatment.

### Disease Severity Scores

Disease severity at the time of the initial DXA scan was evaluated by the same physician (BA) using the Functional Severity Scale (FuSS) [21] and the Brief Ataxia Rating Scale (BARS) [22]. FuSS is comprised of ten functional categories: motor development, cognitive ability, psychological status, expressive speech, balance, oral coordination, frequency of dysautonomic crises, respiratory status, cardiovascular status and nutritional status [21]. Each category is scored from 1 (worst or severely affected) to a maximum 5 (best or no impairment), to a total score of 50.

As balance is an important risk factor for fracture, we examined this component of the FuSS separately. Capability in this category was assessed by the ability to perform various maneuvers at the time of the neurological examination, as described in Table 1 [21].

**Table 1:** The balance score and the Brief Ataxia Rating Scale

	Score	Description
Balance score	1	severe ataxia that prevented independent ambulation and necessitated use of a wheelchair or scooter for mobility
	2	ataxic gait but able to walk with assistance, e.g., use of a walker, cane or someone’s arm
	3	can walk a corridor without a walker but gait is very unsteady. The individual cannot hop or balance on one foot
	4	can walk a corridor without a walker but the gait may be wide-based; cannot balance on one foot but may be able to hop at least one time on either foot
	5	can walk a corridor without support and can balance on at least one foot and can hop
BARS	0–8	Gait
	0–4*	Knee-tibia test (decomposition of movement and intention tremor, left and right scored)
	0–4	Dysarthria
	0–2	Oculomotor abnormalities
	0–4*	Finger-to-nose test (decomposition and dysmetria of the arm and hand, left and right scored)

The balance score is one category of the Functional Severity Scale, and is scored between 1 (worst or severely affected) to 5 (best) [17]. The Brief Ataxia Rating Scale (BARS) has five categories. The accumulative score ranges from 0 (no ataxia) to 30 (most severe ataxia) [18]

*For each side

The severity of ataxia was assessed by the BARS [22]. This scale has five categories, with an accumulative score ranging from 0 (no ataxia) to 30 (most severe ataxia). The scores for the five items are detailed in Table 1. Of note, a higher score in BARS indicates more severe disease, in contrast to higher scores in FuSS and the balance score, which indicate less severe disease.

We assessed ambulation ability using a 4-point scale: 1-constant use of a wheelchair for ambulation; 2-able to ambulate unassisted within the home; 3-able to ambulate up to 100 m unassisted; 4-able to ambulate more than 100 m unassisted***.***

### Bone Mineral Density

BMD was evaluated using DXA (Lunar Prodigy Advance; GE Healthcare, Wisconsin, USA). All the measurements were done at the Sheba Medical Center. In patients aged > 20 years, BMD was measured at the lumbar spine, the femurs and the 33% radius. In patients aged ≤ 20 years, BMD was measured at the lumbar spine and the femurs, and for the total body less head (TBLH). Lumbar and TBLH measurements were not performed in patients who underwent surgical fixation for kyphoscoliosis. BMD measurements were reported as grams per square centimeter (g/cm^2^) and as Z-scores, adjusted for age and sex based on healthy control data. The least significant changes at the 95% confidence levels of the DXA measures at our facility are 0.037 g/cm^2^ at the lumbar spine, 0.025 g/cm^2^ at the dual femur, and 0.043 g/cm^2^ at the 33% radius.

### Trabecular Bone Score

TBS measurements were obtained from DXA spinal images using TBS iNsight Software version 2.1 (Medimaps Group, Geneva, Switzerland). BMD and TBS measurements at the L1–L4 levels were not reported in cases where significant vertebral deformities, such as scoliosis, affected these segments, in accordance with the recommendations of the International Society for Clinical Densitometry (ISCD) [23]. TBS is influenced by age and sex. For patients older than 20 years, TBS Z-scores adjusted for age and sex were provided by the manufacturer. For patients aged 20 years or younger, Z-scores were calculated using reference data from a large study that utilized the same densitometer and TBS software as described above [24]. This reference study provided TBS reference values (mean ± standard deviation) stratified by age and sex. These were based on measurements from 4,127 healthy individuals (2,659 females and 1,468 males), ranging from newborns to 20 years of age. Accordingly, for patients in our cohort aged ≤ 20 years, the TBS Z-score was calculated using the following formula:

$$ {\text{TBS}}\;{\text{Z-score}} = \left( {{\text{TBS}} - {\text{TBS}}_{{{\text{norm}}}} } \right)/{\text{SD}}_{{{\text{norm}}}} $$

where TBS_norm_ and SD_norm_ refer to the mean and standard deviation values from the reference population.

### Anthropometric Measurements

Height and weight were measured just before the DXA scan. Body mass index (BMI) was calculated according to the formula: weight (kg)/height (m)^2^. Height, weight and BMI Z-scores were calculated using age and sex-specific growth data (based on the Centers for Disease Control and Prevention's Year 2000 Growth Charts), which were found adequate also for the Israeli population [25]. For patients older than age 20 years, the height, weight and BMI Z-scores were calculated as if the patients were aged 20 years. The Z-scores were used for further analysis, to avoid age bias.

### Laboratory Analysis

We extracted from patients' medical records the following blood tests that were done in proximity to the time of DXA scans: calcium, phosphorous, alkaline phosphatase, 25-hydroxy vitamin D, parathyroid hormone, C-terminal telopeptides of type I collagen (CTX) and procollagen type I N-terminal propeptide (P1NP). All blood samples were collected in the morning following an overnight fast. Serum CTX and P1NP concentrations were measured on the IDS-iSYS Automated System (Immunodiagnostic Systems, Frankfurt am Main, Germany). Both assays are chemiluminescence immunoassays. The intra- and inter-assay CV for CTX were 2.1–4.9% and 4.7–8.8%, respectively; and the intra- and inter-assay CVs for P1NP were 2.6–3 and 4.2–5.3%, respectively.

### Ethical Considerations

The study was approved by the Institutional Review Board of Sheba Medical Center. Informed consent was waived due to the retrospective design of the study.

### Statistical Analysis

Data were analyzed with the IBM SPSS software version 25 (IBM Corp., New York, USA). Categorical variables were presented as numbers and percentages. Continuous variables were presented as means and standard deviations, or as medians and interquartile ranges, as appropriate. The Student's t-test or Mann–Whitney test was applied to compare patients with and without DXA scans, and between patients with and without a history of fractures. The mean TBS Z-score was compared to the expected value using the one sample t-test. Correlations of BMD and TBS Z-scores with disease severity anthropometric measurements and biochemical parameters were described using Pearson’s correlation test or Spearman correlation. A *p* value < 0.05 was considered statistically significant.

## Results

### Baseline Characteristics

Seventy patients with FD were treated at our institution during the study period. Of these, 41 (21 males, 20 females) underwent at least one DXA scan: 19 patients had one scan, 11 had two scans, 9 had three scans, and 2 had four scans. A comparative analysis revealed no significant differences between patients who underwent DXA scans and those who did not, with respect to: age, sex, and disease severity, as assessed by FuSS scores, balance, and BARS.

The median age at the first DXA scan was 25 years (range 7–47); 14 patients were younger than 20 years. The median age at the last follow up was 32 years (range 12–53). Clinical characteristics, anthropometric measurements and laboratory results of the cohort are presented in Table 2. Seven patients were treated with antiepileptic drugs, three with anti-depressive drugs, 11 with fludrocortisone and 15 with proton pump inhibitors.

**Table 2:** Clinical characteristics, laboratory tests and anthropometric measurements at the first dual energy X-ray absorptiometry scan of 41 patients with familial dysautonomia

Characteristics	Values
Age at the first DXA scan, years	25.0 (15.5–30.5)
Age at the last follow up, years	32.0 (22.5–36.5)
*Gender*	
Females	20 (49%)
Males	21 (51%)
Age at menarche, years	15.3 ± 2.2
Chronic kidney disease	11 (27%)
Scoliosis	31 (76%)
*Disease severity scores*	
Functional Severity Scale	38 (33–42)
Balance score	4 (3–5)
Brief Ataxia Rating Scale score	7 (4–12)
*Laboratory values*	
Hemoglobin, g/dl	11.6 (10.7–12.7)
C-reactive protein, mg/l	4.9 (1.1–28.2)
Calcium, mg/dl	9.5 (9.2–9.8)
Phosphorus, mg/dl	3.8 (3.5–4.5)
Alkaline phosphatase, IU/l	104 (67–178)
25-hydroxy vitamin D, ng/ml	26.9 (20.7–31.5)
Parathyroid hormone, pg/ml	45 (25.2–60.8)
C-terminal telopeptides, pg/ml	1530.6 (825.3–1733.8)
Procollagen type I N-terminal propeptide, ng/ml	135.9 (86.4–290.6)
*Anthropometric measurements*	
Height Z-score	− 2.6 ± 1.5
Weight Z-score	− 3.5 ± 2.2
BMI Z-score	− 2.0 ± 1.7

The values are presented as number (percentile), mean ± standard deviation or median (interquartile range), as appropriate. The range of scores are 10–50 on the Functional Severity Scale and 1–5 on the Balance score. Higher scores indicate a better condition. The range of scores on the Brief Ataxia Rating Scale is 0–30. A higher score indicates more severe ataxia

### Disease Severity and Ambulation

The median scores (interquartile range) for the FuSS, balance, and BARS are presented in Table 2. FuSS scores ranged from 25 to 48. Six patients (15%) score between 25 and 30, 24 patients (58%) scored between 31and 40, and 11 patients (27%) scored between 40 and 48.

The balance scores ranged from 1 to 5. Two patients (5%) had a score of 1, seven patients (17%) had a score of 2, nine patients (22%) had a score of 3, 12 patients (29%) had a score of 4, and 11 patients (27%) had a score of 5.

BARS scores ranged from 0 to 29. Twenty-five patients (60%) had scores between 0 and 10, 13 patients (32%) had scores between 11 and 20, and three patients (8%) had scores between 21 and 29.

Regarding ambulation, one patient required a wheelchair for mobility, eight patients (20%) could ambulate unassisted only at home, and 31 patients (77%) were able to ambulate unassisted for more than 100 m.

### BMD and TBS

BMD and TBS measurements at the first DXA scan are presented in Table 3. A BMD Z-score below − 1 was observed in 50–91% of patients, depending on the measurement site. A BMD Z-score below − 2, which is considered the lower limit of the normal range for age [26], was found in 22–70% of the patients. The mean TBS Z-score was − 1.8 ± 1.6, significantly lower than expected in a healthy population (*p* < 0.01).

**Table 3:** Bone mineral density and trabecular bone score at the first dual energy X-ray absorptiometry scan

Dual energy X-ray absorptiometry	n	Z-score (mean ± SD)	Z-score ≤ − 1 n (%)	Z-score ≤ − 2.0 n (%)
Lumbar spine L1–L4	34	− 1.2 ± 1.5	17 (50%)	8 (24%)
Total body less head	12	− 2.2 ± 1.1	10 (83%)	7 (58%)
Left femoral neck	27	− 1.3 ± 1.1	18 (67%)	6 (22%)
Right femoral neck	26	− 1.2 ± 1.2	15 (58%)	7 (27%)
Bilateral proximal femur	26	− 1.3 ± 1.1	19 (73%)	7 (27%)
33% radius	23	− 2.5 ± 1.0	21 (91%)	16 (70%)
Trabecular bone score Z-score	17	− 1.8 ± 1.6	11 (65%)	7 (41%)

We found significant correlations between BMD and disease severity. Lumbar spine and TBLH Z-scores were correlated with better scores of FuSS (r = 0.451, *p* = 0.007 and r = 0.674, *p* = 0.016, respectively). Left femoral neck, right femoral neck and bilateral proximal femur scores were correlated with better scores of balance, BARS and ambulation ability (Table 4). The third radius Z-score was correlated with better scores for balance (r = 0.518, *p* = 0.011), FuSS (r = 0.410, *p* = 0.05) and the ataxia scale (r = − 0.498, *p* = 0.016).

**Table 4:** Correlations of bone mineral density of the femurs with the Functional Severity Scale (FuSS), balance, ambulation and the Brief Ataxia Rating Scale (BARS)

Z-score		Balance	FuSS score	Ambulation	BARS score
Left femoral neck	r	0.563	0.351	0.572	− 0.469
	p	0.002	0.072	0.002	0.014
	n	27	27	26	27
Right femoral neck	r	.5030	0.274	0.510	− 0.443
	p	0.009	0.176	0.009	0.024
	n	26	26	25	26
Bilateral proximal femurs	r	.6120	0.374	0.627	− 0.470
	p	0.001	0.060	0.001	0.015
	n	26	26	25	26

For the FuSS, balance and ambulation, higher scores indicate a better condition. For the BARS, a higher score indicates more severe ataxia

The L1–4 Z-score was also positively correlated with the height Z-score (r = 0.398, *p* = 0.02) and weight Z-score (r = 0.393, *p* = 0.021). The TBLH Z-score was positively correlated with the height Z-score (r = 0.696, *p* = 0.012), weight Z-score (r = 0.889, *p* < 0.001) and BMI Z-score (r = 0.613, *p* = 0.034).

We did not find correlations of BMD with levels of calcium, phosphorous, vitamin D and parathyroid hormone levels.

### Bone Turnover Markers

The values of P1NP and CTX of 15 patients are presented in Table 5. Ten (67%) patients had CTX above the normal range, and three (20%) had high P1NP. Among those aged > 16 years, CTX and P1NP were negatively correlated to L1–4 Z-scores (r = − 0.681, *p* = 0.03 and r = − 0.700, *p* = 0.02, respectively). We also found negative correlations of CTX and P1NP with FuSS (r = − 0.515, *p* = 0.10 and r = − 0.619, *p* = 0.042, respectively). Accordingly, higher CTX and P1NP levels were correlated to worse disease severity.

**Table 5:** C-terminal telopeptides of type I collagen (CTX) and procollagen type I N-terminal propeptide (P1NP) values of 15 patients with familial dysautonomia

No	Age, years	CTX pg/ml	Normal range	P1NP Ng/ml	Normal range
1	18	**1733.8**	34–635	**144.4**	27–127
2	16	**2142.0**	100–601		59–672
3	10	1530.6	497–2096	412.5	323–1242
4	7	1648.9	588–1832	529.5	307–985
5	10	1685.0	497–2096	723.5	323–1242
6	11	1434.6	762–2351		
7	27	**675.0**	34–635	68.3	27–127
8	47	416.0	38–724	49.0	27–127
9	25	**1981.1**	38–724	**189.2**	27–127
10	27	**1563.5**	38–724	110.0	27–127
11	30	**3400.0**	38–724	**218.9**	27–127
12	28	**800.0**	38–724	87.3	27–127
13	27	**1400.0**	38–724	127.4	27–127
14	27	**1414.4**	38–724	83.7	27–127
15	27	**825.3**	38–724	87.3	27–127

^*^Values above the normal range are shown in bold

### Fractures

At the time of the first DXA scan, 20 patients (49%) reported at least one past fracture. Eleven patients had one fracture, two had two fractures, three had three fractures and four had four fractures. Distribution of fractures by anatomical site is presented in Table 6. Patients with or without fractures did not differ significantly in bone density measurements, TBS z-scores, FuSS score, ataxia scale and ambulation.

**Table 6:** Distribution of fractures by anatomical site in familial dysautonomia cohort

Location	No. of fractures
*Upper limb*	
Metacarpal	2
Phalanges	3
Radius/ulna	10
Humerus	1
*Lower limb*	
Tarsal/metatarsal	2
Phalanges	3
Tibia/fibula	8
Femur	1
*Trunk*	
Pelvis	1
vertebra	1
Not otherwise specified	8
Total	40

### Longitudinal Follow Up

Twenty-two patients had two consecutive measurements of BMD of the bilateral proximal femur. The mean time lapse between the first and last scans was 4.0 ± 2.4 years. The mean BMD Z-score at this site was − 1.3 ± 0.9 at the first scan, and − 1.5 ± 0.9 at the last scan (*p* = 0.043).

### Medical Treatment for Bone Health

Twenty-five patients (61%) took calcium supplements and 32 (78%) took vitamin D. Three patients (7%) were treated with bisphosphonates. Two of the latter were treated with bisphosphonates during young adulthood due to low BMD (Z-scores < − 2) and fractures. The third patient received treatment during adolescence. At age 16 years, she was referred by an orthopedic surgeon for bisphosphonate therapy prior to scoliosis surgery. She received three doses of zoledronic acid, and had no significant adverse effects. Notably, her BMD showed substantial improvement. At baseline, the lumbar spine BMD Z-score was − 4.4, and TBLH BMD Z-score was − 3.9. A follow-up DXA scan performed 2.8 years later revealed marked improvement, with a lumbar spine BMD Z-score of − 1.8 and TBLH BMD Z-score of − 1.7. Following this second scan, the patient underwent C5-L4 posterior spinal fusion.

## Discussion

Our findings of low BMD and a high prevalence of fractures among young patients with FD corroborate previous studies [5, 7]. We also demonstrated, for the first time, low TBS in our patients with FD. BMD was correlated with disease severity, poorer balance, lesser ataxia and lesser ambulation.

A BMD Z-score below − 2, which is considered the lower limit of the normal range for age, was observed in 22–70% of patients, depending on the measurement site. Similar findings were reported in a previous study, in which about 50% of the patients had a Z-score < − 2 [7].

Several factors may affect BMD in individuals with FD. We found correlations of BMD with disease severity (FuSS), balance score, ataxia (BARS) and ambulation. These correlations may suggest that physiotherapy to strengthen muscles, improve balance, and improve components of the ataxia scale may improve BMD in individuals with FD. Our findings are complementary to a study that demonstrated correlations between scoliosis, BMD and muscle strength in individuals with FD [6].

We showed for the first time that TBS, a marker of bone microarchitecture, was lower in individuals with FD than expected in the healthy population. Decreased BMD and TBS may result in increased fracture risk. Indeed, 43% of our patients reported having had at least one fracture, similar to findings in previous studies [5, 7].

Most of our patients had CTX levels above the normal range, and some also had elevated P1NP. This is consistent with a study that reported higher levels of both N-telopeptide cross-linked type 1 collagen (NTx, a marker of bone resorption) and osteocalcin (a marker of bone formation) in individuals with FD compared to controls. We found that both CTX and P1NP correlated negatively with BMD Z-scores at the lumbar spine and with the FuSS score. These findings suggest increased bone turnover in patients with more severe disease, which is associated with lower BMD. Therefore, bisphosphonate treatment should be considered for individuals with FD who have elevated CTX, to improve BMD.

In individuals with FD, the femur is the most reliable site for measuring BMD. In contrast, spine measurements are less dependable for longitudinal follow-up due to spinal deformities, which affect 70–80% of those with FD, and may progress over time. Therefore, we focused on examining longitudinal changes in BMD at the proximal femur. The mean BMD Z-score at this site decreased from − 1.3 ± 0.9 at the initial scan to − 1.5 ± 0.9 at the final scan. The average time interval between the two scans was 4.0 ± 2.4 years.

Most of our patients received calcium or vitamin D supplementation. For 80%, their 25-hydroxyvitamin D level was above 20 ng/ml. These findings reflect awareness of the importance of maintaining appropriate calcium and vitamin D levels in individuals with FD. However, despite the high prevalence of low bone mass and fractures, only three patients (7%) were treated with bisphosphonates.

Scoliosis is very common in the context of FD. About 75% of our cohort had scoliosis. Several studies demonstrated a correlation between low bone mass and scoliosis, for example, among postmenopausal women [27], persons with osteogenesis imperfecta [28], and persons with FD [6]. In the latter, for each unit decrease in the BMD Z-score, the average associated increase in the Cobb angle of the spine was 7.2º [6]. The authors suggested that early diagnosis and treatment of osteoporosis in individuals with FD could help mitigate scoliosis in individuals with FD [6].

The 2019 official position of the International Society for Clinical Densitometry recommended bone health evaluations by DXA scan before orthopedic surgeries, including spine surgery [29]. Peri-operative antiresorptive treatment in osteoporotic orthopedic patients may be effective in reducing postoperative complications [29]. A meta-analysis demonstrated that bisphosphonates treatment was associated with better clinical outcome after spine fusion [30, 31]. To date, the effect of bisphosphonates therapy for fracture prevention, or for lowering the risk of scoliosis progression or peri-orthopedic surgeries has not been evaluated in individuals with FD. Further studies are needed to evaluate the positive effect of bisphosphonate therapy in this population.

Our study has several limitations. First, due to the retrospective design, some pertinent data were unavailable, including serum levels of CTX and P1NP levels, as well as detailed information on the mechanisms underlying the fractures. Additionally, DXA scans were performed as part of routine clinical care, according to physician recommendations and patient preference. Since DXA measurements were obtained using a Lunar device rather than a Hologic device, the recently published TBS reference ranges [20] cannot be applied. TBS values were calculated using an algorithm that applies a soft tissue correction based on BMI. This correction has been developed and validated for use in adults. A software version that accounts for tissue thickness correction in pediatric populations is not currently commercially available [32]. Lastly, due to the relatively small size of the cohort, some correlations did not reach statistical significance.

In conclusion, among our patients with FD who underwent DXA, TBS and BMD were low, and the incidence of fractures was high. We observed significant correlations of BMD with disease severity, balance, ataxia and ambulation. These findings suggest that physiotherapy aimed at improving balance and ambulation could potentially enhance BMD. Additionally, the elevated bone turnover observed in this cohort indicates that bisphosphonate therapy should be considered for patients with FD and high CTX levels. Further longitudinal studies are needed to assess the impact of physiotherapy and bisphosphonates on bone health in FD.

## Data Availability

The datasets generated during and/or analyzed during the current study are available from the corresponding author on reasonable request.

## Abbreviations

FD: Familial dysautonomia
DXA: Dual energy x-ray absorptiometry
BMD: Bone mineral density
TBS: Trabecular bone score
CTX: C-terminal telopeptides of type I collagen
P1NP: Procollagen type I N-terminal propeptide
BMI: Body mass index
TBLH: Total body less head
FuSS: Functional Severity Scale
BARS: Brief Ataxia Rating Scale
